# Toxicological effects of polypropylene microplastics and co-exposure with arsenic in *Drosophila melanogaster*

**DOI:** 10.1080/19336934.2026.2707706

**Published:** 2026-07-30

**Authors:** María Llasbeth Hernández-Calderón, Alondra Gallegos-Moreno, Aneet Yamely Miranda-Camacho, David Ramiro Aguillón-Gutiérrez, Sandra Díaz Barriga-Arceo

**Affiliations:** a Centro de Estudios e Investigaciones Interdisciplinarios, Universidad Autónoma de Coahuila, Saltillo, México; b Laboratorio de Citogenética, Facultad de Estudios Superiores Cuautitlán, Universidad Nacional Autónoma de México, México; c Laboratorio de Bioindicadores, Centro de Investigación y Jardín Etnobiológico, Universidad Autónoma de Coahuila, Viesca, México; d Laboratorio de Toxicología y Genética, Facultad de Estudios Superiores Cuautitlán, Universidad Nacional Autónoma de México, Cuautitlán Izcalli, México

**Keywords:** Microplastics, polypropylene, arsenic, cytotoxicity, fecundity

## Abstract

Microplastics (MPs) are particles smaller than 5 mm that, due to their physicochemical properties, can act as carriers for contaminants such as heavy metals. In this study, we evaluated the toxicological effects of polypropylene microplastics (PP-MPs) and their co-exposure with dimethylarsinic acid (DMA) in the *Drosophila melanogaster* model. Third-instar larvae were exposed to the following experimental groups: negative control, positive control (0.02% H_2_O_2_), cosolvent control (ethanol, 6.94% final concentration), PP-MPs (0.01 g/mL), DMA (0.5 mM), and the co-exposure group (PP-MPs + DMA). Larval-to-adult viability, morphology, tissue damage, and fecundity were assessed. PP-MPs alone did not significantly affect larval-to-adult viability, the incidence of morphological abnormalities, or fecundity; however, evidence of tissue damage was observed. In contrast, co-exposure suggested an antagonistic effect on larval-to-adult viability and tissue damage, while indicating a synergistic effect on fecundity and the incidence of abnormalities. These findings contribute to a better understanding of the biological effects associated with co-exposure to microplastics and arsenic in an *in vivo* model.

## 1. Introduction

Microplastics (MPs) are plastic particles smaller than 5 mm [1,2]. MPs can originate from primary sources, where they are intentionally manufactured as raw materials for various products, or from secondary sources, which result from the fragmentation of larger plastic debris through UV radiation, wave and wind abrasion, hydrolysis, and thermal or microbial degradation [3–5].

Human exposure to microplastics is virtually unavoidable due to their ubiquitous distribution in the environment and consumer products. Such exposure can occur through ingestion of contaminated food and beverages [6–13], inhalation of airborne microplastics [14,15], and dermal contact via personal care products, cosmetics, textiles, and dust [16]. Once considered inert particles, microplastics are now recognized as potentially harmful to organisms, as they can reach tissues such as blood, placenta, kidneys, liver, and lungs, as well as biological fluids including breast milk [17–20]. The chemical properties of these plastic particles, along with their additives, can induce oxidative stress, cytotoxicity, and genotoxicity. Their persistent nature, coupled with the lack of enzymatic systems in organisms capable of degrading them, hinders their elimination, leading to chronic inflammation that increases cancer risk. Their involvement has also been proposed to contribute to the rising incidence of immune and neurodegenerative diseases [21]. Moreover, they may serve as vectors for microorganisms and/or toxicants such as heavy metals [22–24].

Over the past decade, the fruit fly (*Drosophila melanogaster*) has become an important model organism for studying the nanotoxicity of plastic particles, nanomaterials, and heavy metals [25–30]. To date, several types of microplastics have been investigated in *D. melanogaster*, including polystyrene (PS) [31–34], polyethylene (PE) [35], polyvinyl chloride (PVC) [35], polyethylene terephthalate (PET) [36–39], polyamide (PA) [40], polypropylene (PP) [41], polytetrafluoroethylene (PTFE) [42], and fluorescent green plastic particles (FMG-Green) [43]. These studies have demonstrated that both particle size and concentration are critical variables when assessing the toxic effects of these contaminants in flies.

Although nanoplastics exhibit a greater capacity for internalization in *D. melanogaster* and may be more numerous due to fragmentation processes, microplastics constitute the fraction most widely detected and characterized in the environment. Therefore, this study prioritized their use, employing particles with variable sizes and shapes to better approximate realistic exposure scenarios, where such materials rarely occur in uniform forms. Within this same framework of environmental and biological relevance, dimethylarsinic acid (DMA) was selected as the arsenic species due to its metabolic and anthropogenic origin. Although it was initially considered less toxic than inorganic arsenic and, consequently, has been studied less, its cytotoxic and genotoxic potential is now recognized [44], highlighting the importance of evaluating its interaction with microplastics.

In this context, regarding the interaction of microplastics with heavy metals in *D. melanogaster*, the impact of co-exposure to polystyrene – cadmium and polystyrene – silver [28] has been reported. However, to date, no studies have evaluated the effect of co-exposure to polypropylene and arsenic in this model, despite polypropylene being one of the most widely produced plastics worldwide [45] and arsenic being a toxicologically relevant metalloid due to its widespread environmental presence [46]. This makes interactions between them highly likely across various ecosystems, although their impact on different species, including humans, remains poorly understood. For this reason, the aim of this study was to evaluate the toxic effects of polypropylene microplastics and their co-exposure to the organic arsenic salt, dimethylarsinic acid (DMA), on viability, morphology, *in vivo* cytotoxicity, and fecundity in *D. melanogaster*.

## 2. Materials and methods

### 2.1. Biological material

All experiments were conducted using the wild-type strain of *Drosophila melanogaster* (^*+/+*^) obtained from the Fly Bank of the Facultad de Ciencias, Universidad Nacional Autónoma de México (UNAM), and adapted for several generations to the conditions of the Cytogenetics Laboratory at the Facultad de Estudios Superiores Cuautitlán. For adult maintenance, flies were reared on a yeast-based culture medium (cornmeal, 17.5 g; sugar, 11.6 g; yeast, 11 g; carrageenan, 1.6 g; gelatin, 0.83 g; propionic acid, 0.6 mL; nipagin 12%, 0.6 mL; and H_2_O, 208.3 mL). They were maintained at 25 ± 2°C, 30–40% relative humidity, and a 12 h light/dark cycle. For obtaining third-instar larvae, the culture medium was prepared as described above, and 20 males and 20 females were crossed. Adults were discarded after 48 h, and 72-h-old larvae were collected (L3).

### 2.2. Microplastics

#### 2.2.1. Preparation of polypropylene microplastic suspension (PP-MPs)

The suspension of polypropylene microplastics was prepared following the methodology reported by Tang et al. (2023) [41], with the following modifications: isotactic polypropylene pellets (Sigma-Aldrich 428,116) were ground in an electric mill, sieved using mesh #200 (74 µm), and kept under agitation (PolyScience Shaking Water Bath System, PolyScience, Niles, IL, USA) at room temperature for 48 h at 192 ± 8 cpm. The sieved polypropylene was collected and suspended in 50% ethanol at a concentration of 1 g in 12 mL. The suspension was maintained under magnetic stirring for 2 h and subsequently sonicated at 35 kHz (Transsonic T 420, Elma, Singen, Germany) for 30 min at room temperature.

#### 2.2.2. Characterization of microplastics

Characterization was performed at the Microscopy Laboratory of the Centro de Física Aplicada y Tecnología Avanzada (CFATA), UNAM, using scanning electron microscopy (Hitachi SU 8230, Hitachi, Tokyo, Japan) at 1 kV and different magnifications. The size of polypropylene particles was determined using the Max Feret parameter, defined as the maximum distance between two parallel lines tangent to the contour of a particle. The zeta potential of PP-MPs was determined using a Zetasizer Nano ZS90 (Malvern Panalytical, UK) based on electrophoretic light scattering under controlled conditions. Measurements were performed to assess particle surface charge and colloidal stability. Polypropylene was identified using Fourier-transform infrared (FTIR) spectroscopy (MB 3000 Series, ABB, Canada) under standard operating conditions. Spectra were acquired in transmittance mode over the 3700–500 cm^−1^ range, with a resolution of 1 cm^−1^ and 100 scans.

### 2.3. Experimental groups

Instant *Drosophila* medium (TM MEDIA, TM 1170) was prepared as per the manufacturer’s instructions with a 10% reduction in the recommended medium weight. Once cooled to 50°C, the corresponding treatment for each experimental group was added as follows: negative control, sterile water for injection (PiSA); positive control, hydrogen peroxide (J.T. Baker, 2186–01) 0.02% (final concentration); co-solvent control, ethanol (tecsiquim, ET001) 50% (final ethanol concentration in the culture medium:6.94%); PP-MPs (ethanol, 6.94%; PP-MP_S_ 0.01 g/mL, final concentrations in the culture medium); DMA (Sigma-Aldrich, C0250) 0.5 mM (final concentration); and co-exposure PP-MP_S_ [0.01 g/mL] + DMA [0.5 mM]. A total of 10 mL of culture medium was dispensed into sterile culture vials and allowed to solidify at room temperature. Three replicates per group were performed, each containing 50 third-instar larvae.

### 2.4. Presence of microplastics in the intestine of Drosophila melanogaster

To confirm larval ingestion of PP-MPs, their presence in the intestine was identified using the Nile Red staining protocol reported by Alaraby et al. (2023) [47], with slight modifications. The PP-MP suspension [0.01 g/mL] was centrifuged at 14,000 rpm for 30 min, the upper supernatant was collected, and the MPs were transferred into a new 1.5 mL tube. One millilitre of Nile Red (Sigma-Aldrich, 19,123) (0.5% in DMSO (Sigma-Aldrich, D8418)) was added, and the suspension was kept under constant agitation for 24 h at 185 cpm. Stained PP-MPs were washed twice with ethanol in PBS 1X (pH = 7.4). The stained PP-MP_S_ were mixed with standard *Drosophila* medium (500 µg/g food), and 10 mL of the mixture was transferred into a culture vial. After solidification, 50 larvae (72 h old) were added and fed for 48 h. Afterwards, larval intestines were dissected under a stereomicroscope and analysed by epifluorescence microscopy (ACCU-SCOPE EXC-350, ACCU-SCOPE Inc., Commack, NY, USA) at 4× and 10× with excitation/emission filters of 540–560 nm and 580–620 nm, respectively. All procedures were performed under minimal direct light exposure. Image processing was conducted with ImageJ version 1.54d (National Institutes of Health, USA), applying the manual threshold adjustment tool to enhance signal detection and facilitate the identification of PP-MP_S_ [48].

### 2.5. Larval-to-adult viability and morphological evaluation

Larval-to-adult viability was determined as the total number of living adults emerged per experimental group. After eclosion, adults were anesthetized with anhydrous ethyl ether, and their morphology was examined under a stereomicroscope (VELAB STEREO VE-S6, VELAB Co., Pharr, TX, USA) to identify morphological abnormalities. Photographs were taken using a VELAB VE-LX1800 camera (VELAB Co., Pharr, TX, USA) and ToupView software (TOUPTEK PHOTONICS, version x64, 4 November 21973.20230107, ToupTek, Hangzhou, China).

### 2.6. Evaluation of tissue damage by trypan blue exclusion

Tissue damage by trypan blue exclusion was assessed following the protocol of Priyadarsini et al. (2020) [49], as described next: 30 third-instar larvae were placed in culture vials containing the different treatments and fed for approximately 16 h. After exposure, larvae were recovered, washed in PBS 1X, and transferred into 1.5 mL tubes containing 0.5 mL of 0.2% trypan blue (Sigma-Aldrich, T8154), where they remained for 30 min at room temperature. The dye was then removed, and larvae were washed three times with PBS 1X for 10 min each. Trypan blue uptake was used as an indicator of loss of epithelial membrane integrity and, therefore, as a semi-quantitative marker of tissue damage. This approach is commonly employed *in vivo* larval models, including both larval and adult stages, to assess epithelial barrier disruption and tissue integrity. In the present study, staining was evaluated to identify damage in the intestinal tissue as well as in other structures exposed to the evaluated xenobiotics. Staining intensity was evaluated under a stereomicroscope using the following scoring system: no staining = 0; light staining = +; faint blue staining across the tissue = ++; strong blue staining across the tissue = +++. Each larva was assigned to the corresponding damage category, and the percentage of larvae within each category was subsequently calculated and plotted.

### 2.7. Fecundity analysis of exposed females

Third-instar larvae were exposed to the different experimental groups. Once they reached the mature pupal stage, virgin females were collected and, after 24 h, crossed with non-exposed males. Embryo collection was performed according to the protocol of Sánchez et al. (2023) [50]. Agar plates of 6 cm diameter were used and crosses of 10 non-exposed males with 10 exposed females were established in embryo collection chambers. The average number of embryos per female was recorded on days 1, 7, 14, 21, and 28, and all experiments were conducted in triplicate.

### 2.8. Statistical analysis

All statistical analyses were performed using SPSS version 29.0.2.0. Data are presented as mean ± standard error (SE). For variables with normal distribution, one-way ANOVA was applied, followed by Tamhane’s post hoc test or Student’s *t*-test. For variables with non-normal distribution, the Kruskal – Wallis test was used followed by Dunn’s post hoc test with Bonferroni correction. Statistical significance was set at *p* < 0.05.

## 3. Results

### 3.1. Characterization of microplastics

**Figure 1.**
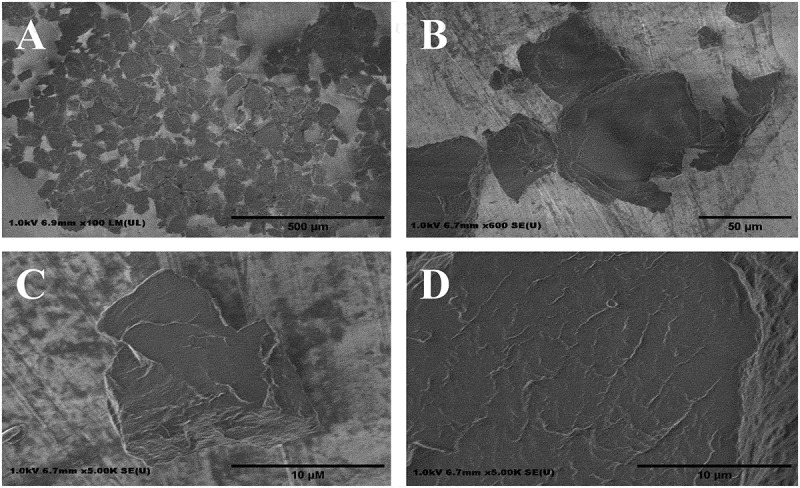
Analysis of polypropylene microplastics by scanning electron microscopy. (A) A heterogeneous population of PP-MPs was observed, exhibiting variability in both size and morphology; (B) and (C) polyhedral shapes of PP-MP_S_; (D) surface of a polypropylene microparticle showing that it is not an aggregate of smaller particles.

Scanning electron microscopy characterization revealed a heterogeneous population of polypropylene microparticles in both shape and size (Figure 1). The PP-MPs exhibited polyhedral shapes, with an average particle size of 38.69 ± 20.52 µm (range: 7.52–84.91 µm; *n* = 100). The particle size distribution is shown in Figure S1, revealing a broad and heterogeneous distribution, with most particles concentrated between approximately 17–27 µm, and a slight secondary peak around 37–47 µm. The PP-MP_S_ exhibited a zeta potential of −72.43 mV in a hydroalcoholic medium, suggesting a strongly negative surface charge and high colloidal stability under the experimental conditions. FTIR analysis confirmed the identity of polypropylene based on its characteristic spectral bands (Figure S2).

### 3.2. Presence of microplastics in the intestine of Drosophila melanogaster

**Figure 2.**
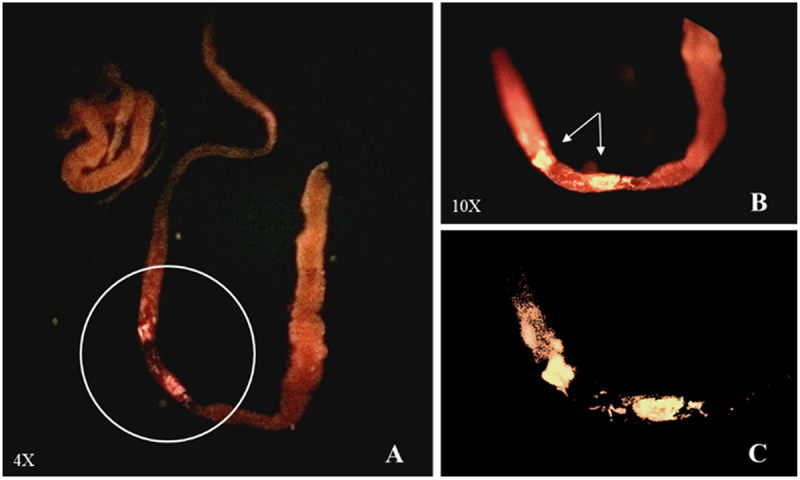
Presence of polypropylene microplastics in the intestine of *Drosophila melanogaster*. (A) Larval intestine of *D. melanogaster*, with the circle indicating the region containing the larger PP-MPs, viewed at 4×; (B) close-up at 10× of the intestinal region containing the larger polypropylene microplastics, indicated by arrows; (C) image processing to reduce tissue-inherent background fluorescence, highlighting the polypropylene microplastics.

To determine whether the larvae were ingesting the PP-MP_S_ and to identify the organs in which they were present, third-instar larvae were fed Nile Red – stained PP-MP_S_ mixed with standard food. Dissection of the larvae and analysis by epifluorescence microscopy showed the presence of PP-MP_S_ in the larval intestine (Figure 2). It should be noted that tissues can exhibit autofluorescence; therefore, image processing is recommended to remove background fluorescence inherent to the tissue. In this study, image processing was performed using ImageJ.

**Figure 3.**
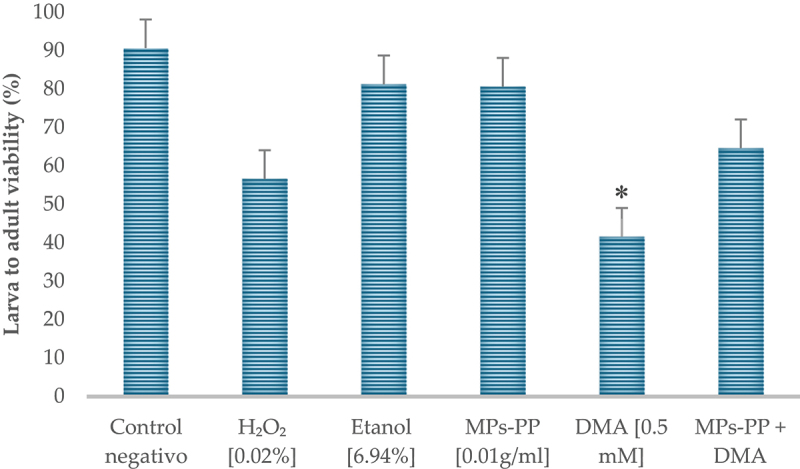
Larval-to-adult viability in the different experimental groups. data are presented as mean ± SE (*n* = 50). Significant differences compared to the negative control, ANOVA followed by Tamhane’s test, *(*p* < 0.05).

Larval-to-adult (L3–adult) viability was assessed seven days after larval exposure to the different treatments, and the life cycle was monitored every 24 hours. As shown in Figure 3, the experimental groups exhibiting larval-to-adult viability below 80% were those exposed to hydrogen peroxide, arsenic, and PP-MPs + DMA. However, only the DMA exposed group showed statistically significant differences compared to the negative control (ANOVA followed by Tamhane’s test, *p* < 0.05).

**Figure 4.**
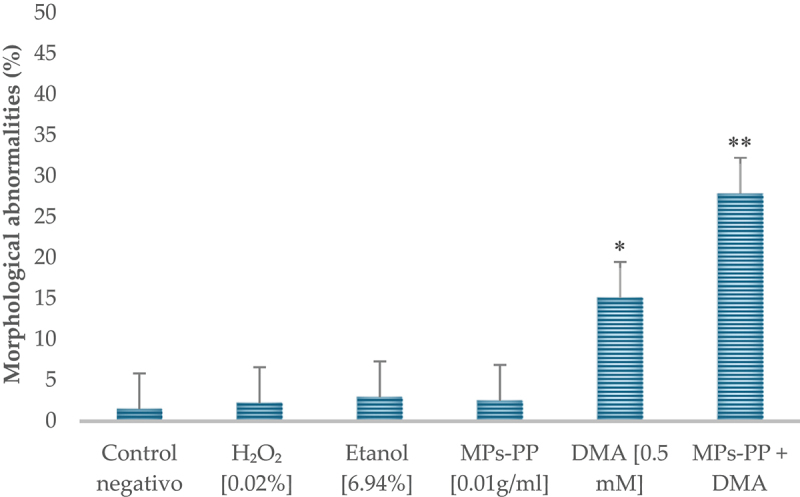
identified in the different experimental groups. Data are presented as mean ± SE, with sample size (*n = 50*), which varied according to the observed viability percentage in each experimental group. Statistically significant differences compared to the negative control, Kruskal–Wallis test followed by Dunn’s post hoc test with Bonferroni correction, *p < 0.05, **p < 0.01.

**Figure 5.**
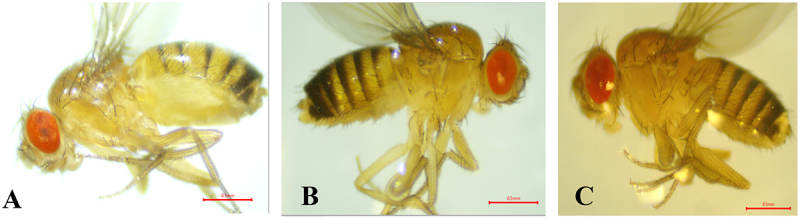
Abdominal abnormalities in female *D. melanogaster* exposed to DMA and PP-MPs + DMA. (A) Control; (B) Females exposed to DMA from L3; (C) females exposed to PP-MPs + DMA from L3. Views at 4x. Scale bar = 0.5 mm.

During morphological analysis, adult flies with abnormalities were identified, mainly in the legs and wings, in the groups exposed to H_2_O_2_, ethanol, and PP-MP_S_. However, the experimental groups that showed significant differences compared to the negative control were those exposed to DMA (*p* < 0.05) and the co-exposure group to PP-MPs + DMA (*p* < 0.01) (Figure 4). In these groups, the main abnormalities were observed in females and consisted of differences in abdominal size, which resulting in flies appearing disproportionate relative to their thorax and head size (Figure 5).

**Figure 6.**
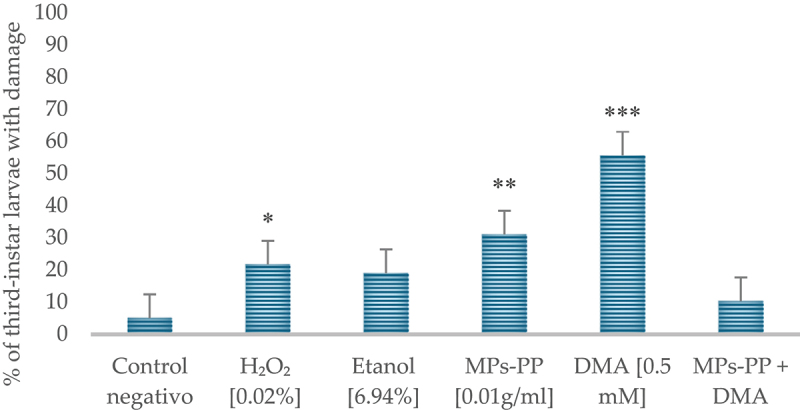
Assessment of *tissue damage* in third-instar larvae of *Drosophila melanogaster*. The percentage of larvae exhibiting “+” grade tissue damage was plotted, as determined using a semi-quantitative scoring system based on staining intensity data are presented as mean ± SE (*n* = 30). Statistically significant differences compared to the negative control, Kruskal–Wallis test followed by Dunn’s post hoc test with Bonferroni correction, *p < 0.05, **p < 0.01, ***p < 0.001.

For the assessment of tissue damage, third-instar larvae were exposed to each treatment for 16 hours, a period selected to ensure adequate ingestion of the test compounds while maintaining larval viability and allowing the detection of sublethal tissue damage. During this time, larvae remained active and feeding. Microscopic analysis revealed that the induced damage was low and localized (+, according to the scale), primarily affecting the tegument. In the groups exposed to DMA and PP-MP_S_, damage was also observed in the intestine (+, according to the scale), suggesting a potential compromise of epithelial barrier integrity (Figure S3). Significant differences compared to the negative control were found in the groups exposed to H_2_O_2_ (*p* < 0.05), PP-MPs (*p* < 0.01), and DMA (*p* < 0.001) (Figure 6).

**Figure 7.**
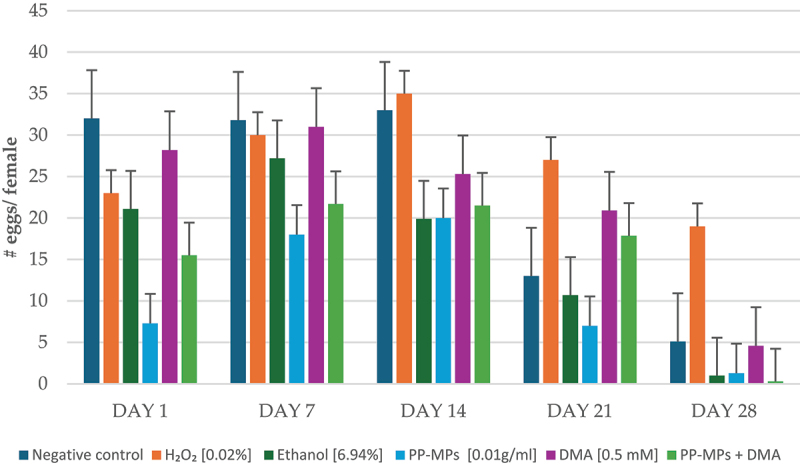
Fecundity of *Drosophila melanogaster* exposed to the different experimental groups. Data are presented as mean ± SE (*n* = 10). No statistically significant differences were found between the groups compared to the negative control (kruskal-Wallis test followed by Dunn’s post hoc test with Bonferroni correction, *p > 0.05*).

The number of eggs laid per female was determined on days 1, 7, 14, 21, and 28. As shown in Figure 7, in most experimental groups, the peak egg-laying occurred on day 14, except for the DMA group, which peaked on day 7. From day 21 onward, egg production began to decline and was nearly zero by day 28. Notably, female mortality started to occur from day 21.

## 4. Discussion

In the present study, the toxic effects induced by PP-MPs and their co-exposure with an organic arsenic salt (DMA) were evaluated using *Drosophila melanogaster* as a model. The effects of these environmental contaminants on larval-to-adult viability, fly development, larval tissue damage, and female fecundity were evaluated.

Fluorescence microscopy analysis confirmed the presence of PP-MPs in the midgut of third-instar larvae. Exploratory examination of tissues, such as larval glands and the brain, did not reveal the presence of microplastics. However, this approach does not allow a direct or quantitative assessment of particle translocation; therefore, migration of smaller particles that may not have been detected by this technique cannot be ruled out. Nevertheless, given the particle size used in this study, translocation across the intestinal epithelium is considered unlikely, as this process has been primarily described for smaller particles. Consequently, the observed effects are mainly interpreted as the result of local interactions at the intestinal level, possibly associated with oxidative stress processes; however, this mechanism was not assessed in the present study and should be addressed in future research.

From an experimental perspective, it is important to consider that ethanol may exert biological effects at certain concentrations; however, no significant differences were observed between the co-solvent control (6.94%) and the negative control across any of the evaluated endpoints. These results suggest that, under the experimental conditions employed, ethanol did not contribute significantly to the responses observed. Therefore, the detected effects can be primarily attributed to PP-MPs in systems in which ethanol was used as the co-solvent.

Larval viability assays indicated no significant toxicity to L3-to-adult viability in *D. melanogaster*, as no differences were observed compared to the negative control. This low impact on viability across different developmental stages has been reported for polystyrene microplastics [28,51] and nanoplastics (particles ranging from 1 nm to 1 μm [52,53] such as polystyrene [26,27,54], PET [30,47], polypropylene [55], and even bioplastics like polylactic acid [29].

Although micro/nanoplastics do not appear to significantly affect the viability of *Drosophila* at any developmental stage, some studies have reported alterations in the duration of life cycle stages. For instance, PVC microplastics shorten the larval and pupal stages [35]; PET reduces female survival at high concentrations [38] while extending male lifespan [36]; polystyrene decreases adult survival [33]; and fluorescent green microparticles reduce overall lifespan [43]. Furthermore, polystyrene nanoplastics can delay pupation and eclosion by up to 24 hours [56], and PET decreases survival in a concentration-dependent manner [57]. Scientific evidence suggests that smaller particles and higher exposure concentrations increase the observed developmental and viability damage, likely due to greater bioavailability, translocation to other organs and tissues, and accumulation within the fly.

As expected, DMA significantly decreased larval-to-adult viability, even below the positive control (H_2_O_2_), resulting in mortality at early developmental stages, as evidenced by dead larvae in culture vials, and to a lesser extent at later stages, with non-viable pupae observed. This observation aligns with findings reported by Meyer et al. (2014) [58], who observed reduced pupation and eclosion rates following exposure to arsenolipids, and by Beamish et al. (2021) [59], who demonstrated that arsenite delays pupation and induces larval lethality. The concentration of DMA used in this study was taken from Rizki et al. (2006) [60], where it was employed in *Drosophila melanogaster* experimental assays and corresponds to a biologically active concentration that induces measurable toxic effects.

Interestingly, co-exposure to PP-MPs + DMA did not result in significant differences compared to the negative control, suggesting an antagonistic interaction between PP-MPs and arsenic, which appears to reduce the damage caused by arsenic alone while also slightly decreasing the cytotoxic effects of PP-MPs. Similar antagonistic relationships between micro/nanoplastics and heavy metals have been reported for polystyrene-silver combinations [28].

Morphological analysis showed that PP-MPs induced abnormalities in legs and wings, although the frequency was not significant compared to the negative control. Few studies have evaluated morphological alterations in adult flies exposed to micro/nanoplastics, and results are variable regarding the teratogenic potential of these xenobiotics. PET microplastics have been reported to induce abnormalities in the abdomen, eyes, and wings, whereas polystyrene micro/nanoplastics may not induce morphological changes [56,61], or may affect the abdomen, wings, head, and thorax [51], and even cause sexual dimorphic alterations in heart size and function [62].

Regarding arsenic, the main morphological abnormalities observed in this study were abdominal, occurring exclusively in females. Stereoscopic microscopy revealed smaller abdomens in some females, causing adults to appear disproportionate relative to the head and thorax. Since female abdominal volume is largely occupied by the ovaries, this alteration could be related to arsenic-induced ovarian structural damage, although further studies are required to confirm this. The literature reports that arsenic impacts fly behaviour, and in other biological models, including hamsters, chickens, mice, rats, and salamanders, various morphological alterations have been documented, demonstrating the teratogenicity of this metalloid [63–65].

In the PP-MPs + DMA co-exposure group, the same abdominal abnormalities were observed as in the arsenic-only group and remained female-specific. Statistical analysis indicated that while there were significant differences compared to the negative control, no significant differences were found between the DMA and PP-MPs + DMA groups, suggesting that the effect is primarily attributable to arsenic, with the increased percentage of abnormalities potentially reflecting improved viability due to the microplastic – metalloid interaction. To date, no studies have evaluated morphological alterations in *D. melanogaster* after co-exposure to a microplastic and a metal/metalloid, highlighting the novelty of this work in toxicological assessment.

Tissue damage analysis demonstrated that both PP-MPs and DMA induce damage to the tegument and midgut of third-instar larvae, even more extensively than the positive control, which may cause lipid peroxidation as insect cuticles contain waxes, fatty acids, and sterols [66]. Damage to the midgut is consistent with epifluorescence microscopy observations, showing PP-MPs present in the intestine.

Similar effects have been reported for other micro/nanoplastics such as PET, polystyrene, and polylactic acid, which also cause intestinal barrier damage, enterocyte necrosis, apoptosis, inflammation, interaction with symbiotic gut bacteria, and increased expression of genes such as *drice* and *p53*, indicative of cellular damage response [30,33,39,56,67].

Tegument damage may result partly from oxidative stress induced by microplastics and reactive oxygen species generated by arsenic, and partly from mechanical friction between larvae and PP-MPs during locomotion. Notably, the cytotoxic effect of PP-MPs and DMA was reduced during co-exposure, suggesting an antagonistic interaction between the two contaminants.

Fecundity analysis showed that both PP-MPs and PP-MPs + DMA affected female reproductive health, with a decrease in egg-laying observed, although it was not statistically significant. The literature indicates that nanoplastics, particularly polystyrene particles smaller than 100 nm, can migrate to and accumulate in the ovary and oocyte, causing reproductive toxicity at the molecular level in flies [67,68]. Regarding microplastics, PET particles have been reported to cause ovarian cellular damage and reduce reproductive health, as evidenced by decreased fertility [39], while fluorescent green particles reduce ovary size, egg-laying, and alter the expression of genes related to genotoxicity and inflammation [43].

These findings emphasize the importance of particle size in micro/nanoplastics when studying fecundity. In the present study, the large particle size likely explains the reduction in egg-laying due to ovarian structural damage, consistent with the abdominal abnormalities observed in DMA and PP-MPs + DMA groups, without affecting overall physiological hypothesis, which requires experimental confirmation, as this study did not directly assess it.

Although no significant differences in fecundity were observed among the different evaluated systems, it is important to consider that fecundity was calculated based exclusively on surviving females at the different sampling days. The mortality recorded on days 14 and 21 may introduce a survivorship bias, as the analyses are restricted to individuals who survived the evaluation period. This may influence estimates of reproductive performance without necessarily reflecting detectable differences under the experimental conditions assessed.

One of the main limitations of this study is the use of a single exposure concentration. Although this provides an initial perspective on understanding the behaviour of these contaminants in a terrestrial model, it does not allow the establishment of a concentration-response relationship. Additionally, the absence of molecular and mechanistic analyses limits the understanding of the mechanism of action of these contaminants, such as alterations in signalling pathways, gene expression, or enzymatic activity. Furthermore, computational chemistry studies evaluating the interactions between polypropylene microplastics and DMA are lacking, which could help predict affinities, biomolecule binding, and potential changes in their toxicodynamic.

Overall, these limitations highlight the need for future studies to further optimize experimental conditions, including validating the positive control to ensure a robust biological response in the assessment of viability and morphology. In addition, further research should focus on oxidative stress responses, gene expression profiling, and apoptosis-related pathways, as well as assessing particle biodistribution, tissue-specific interactions, and potential translocation across biological barriers. Finally, toxicokinetic and toxicodynamic approaches will be essential to achieve a more comprehensive understanding of the behaviour of these contaminants, particularly under co-exposure conditions.

### 4.1. Conclusion

In conclusion, individual exposure to PP-MPs induced tissue damage in third-instar *D. melanogaster* larvae, without significantly viability or development, and with a slight reduction in fecundity. Co-exposure with arsenic modified the observed toxic effects compared to single exposure, suggesting potential interactions between these contaminants under the evaluated conditions. However, the underlying mechanisms remain unclear; therefore, further studies are required to determine whether these effects involve alterations in toxicokinetic and toxicodynamic processes. This study highlights the need for additional research to better understand the behaviour of these contaminants in an *in vivo* system.

## Supplementary Material

Supplemental Material

## Data Availability

The authors agree to make the data and materials supporting the results presented in this manuscript available upon reasonable request. Requests for access should be directed to the corresponding author via the email address provided.
