# Supplementary material for: Toxicological effects of polypropylene microplastics and co-exposure with arsenic in Drosophila melanogaster
Source: Fly (Austin). 2026 Jul 30;20(1):2707706. doi: 10.1080/19336934.2026.2707706 (PMC13432825; doi:10.1080/19336934.2026.2707706)
Supplement: Supplemental Material [file KFLY_A_2707706_SM6170.docx]

**SUPPLEMENTARY MATERIAL**


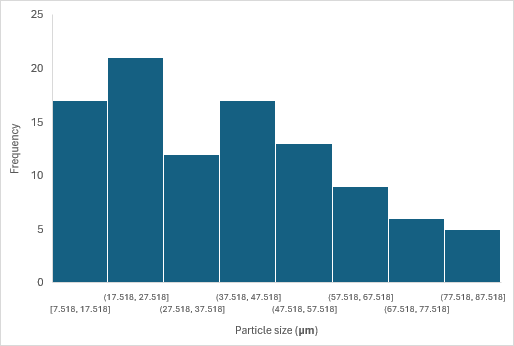


Fig 1S. Size distribution histogram of polypropylene microplastics (PP-MPs).

The graph shows the frequency of particles according to their measured size range (n=100), illustrating the heterogeneity of the sample in terms of particle dimensions. Data are presented as the number of particles within each size class.

Fig 2S. ATR-FTIR spectrum of polypropylene (PP) microplastics recorded in the 3700–500 cm⁻¹ range. The spectrum shows characteristic absorption bands at 2916 cm⁻¹ (C–H stretching vibrations), 1458 cm⁻¹ (CH₃ asymmetric bending), 1373 cm⁻¹ (CH₃ symmetric bending), 1041 cm⁻¹ (skeletal C–C stretching and CH deformation modes), and 972 cm⁻¹ (CH₃ rocking vibration associated with the crystalline phase of isotactic polypropylene), confirming the identification of PP.


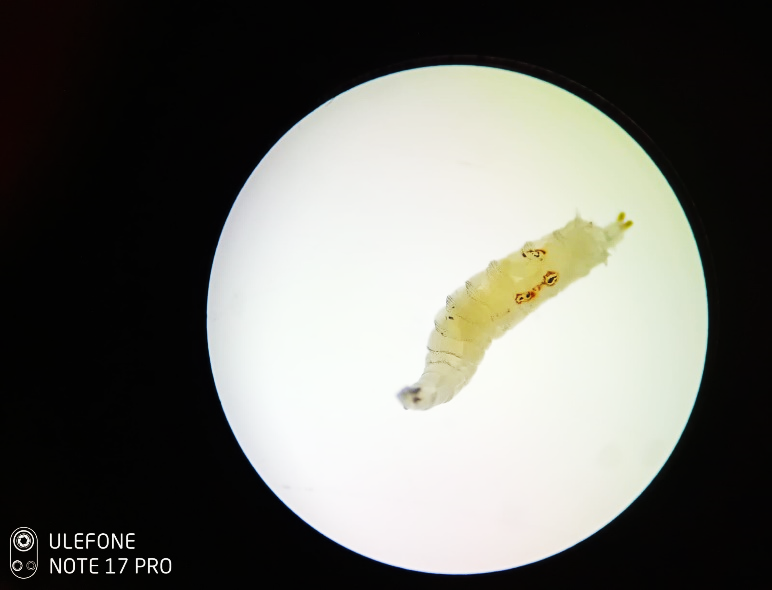

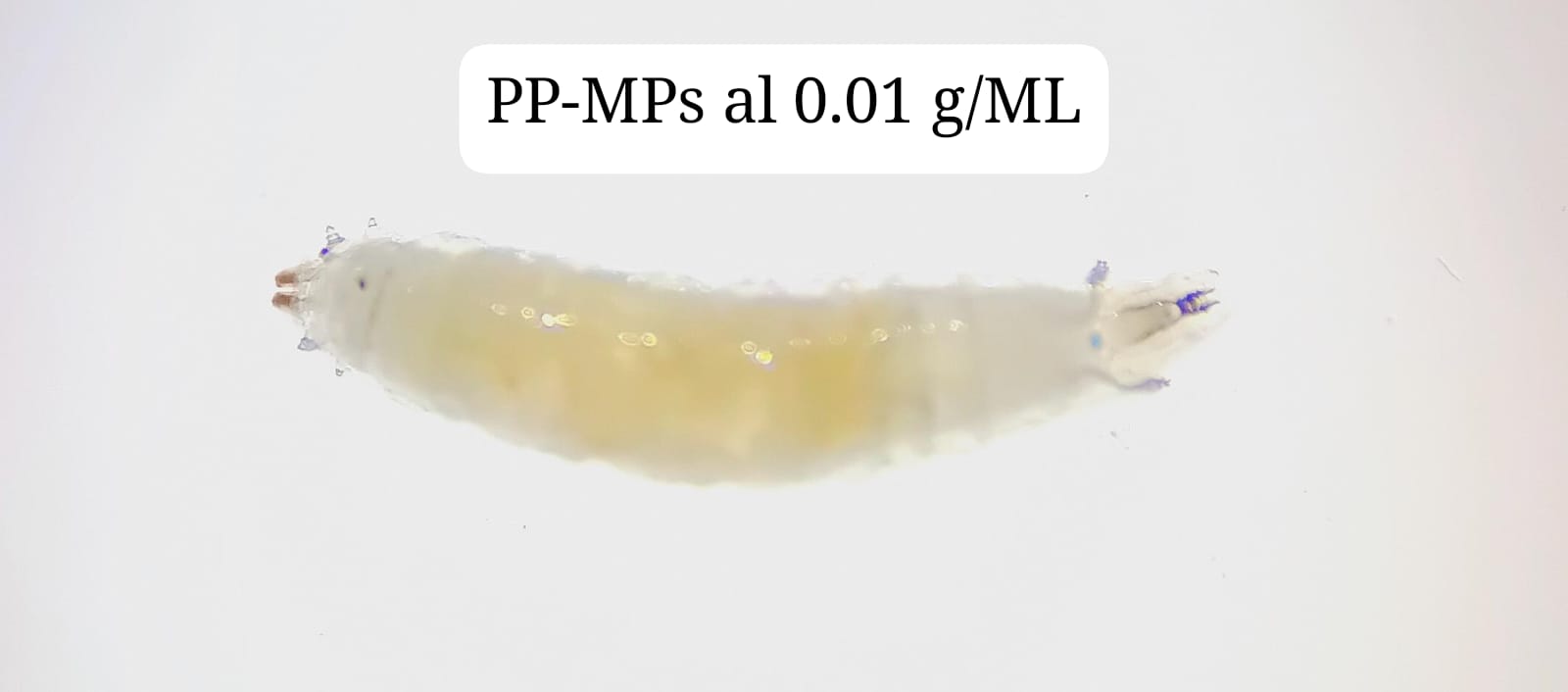

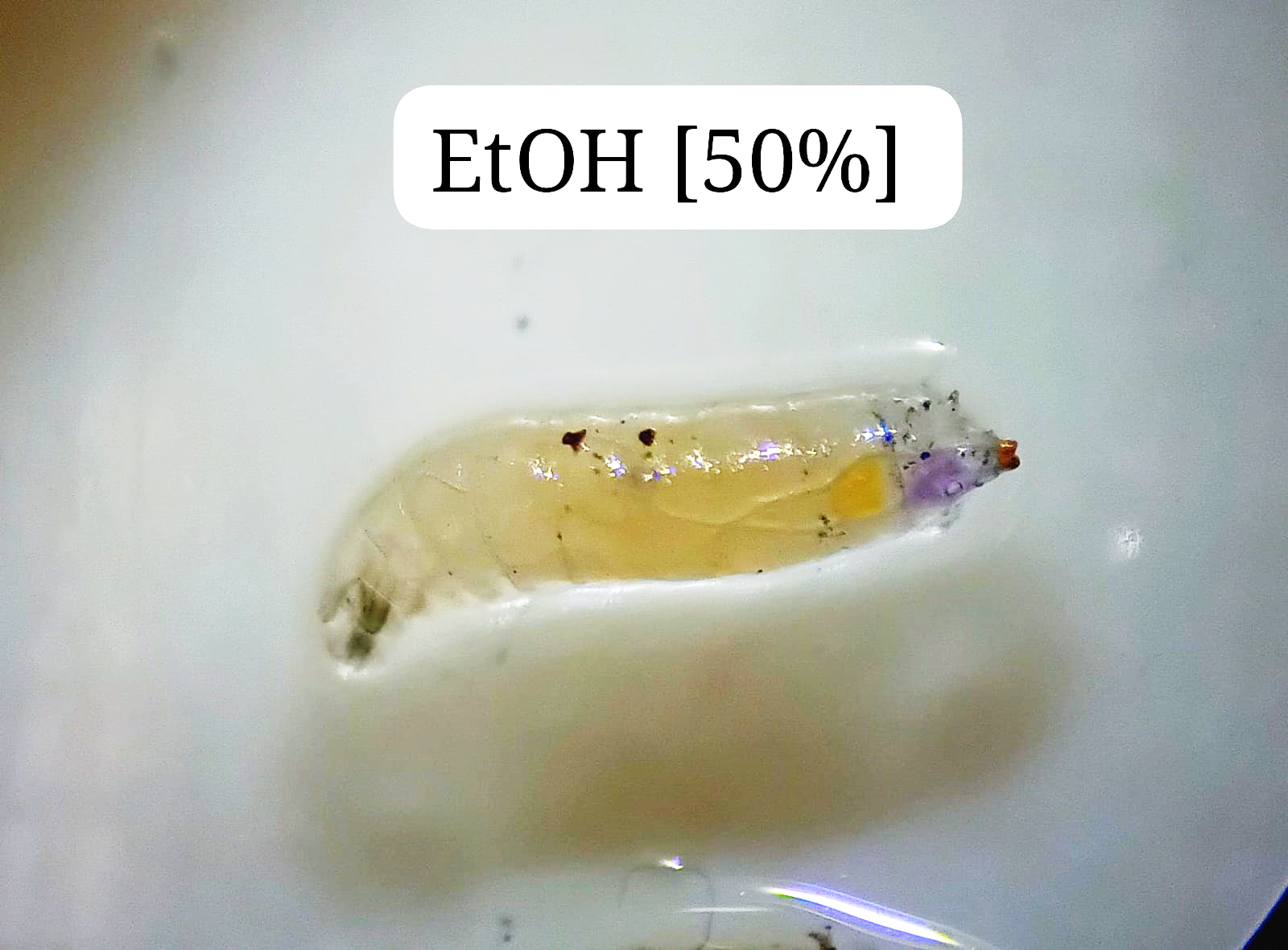

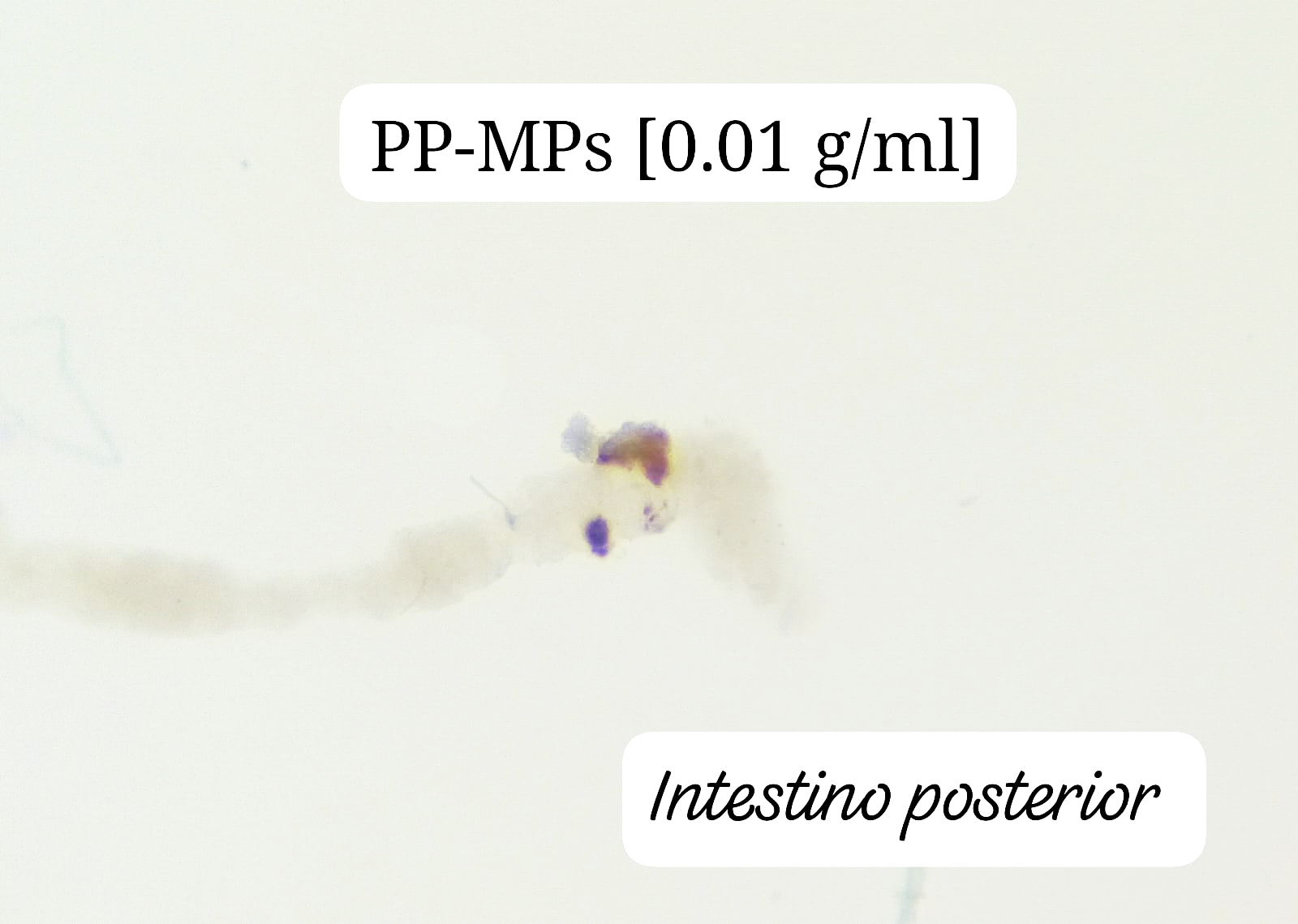


**B**

**C**

**D**

**E**


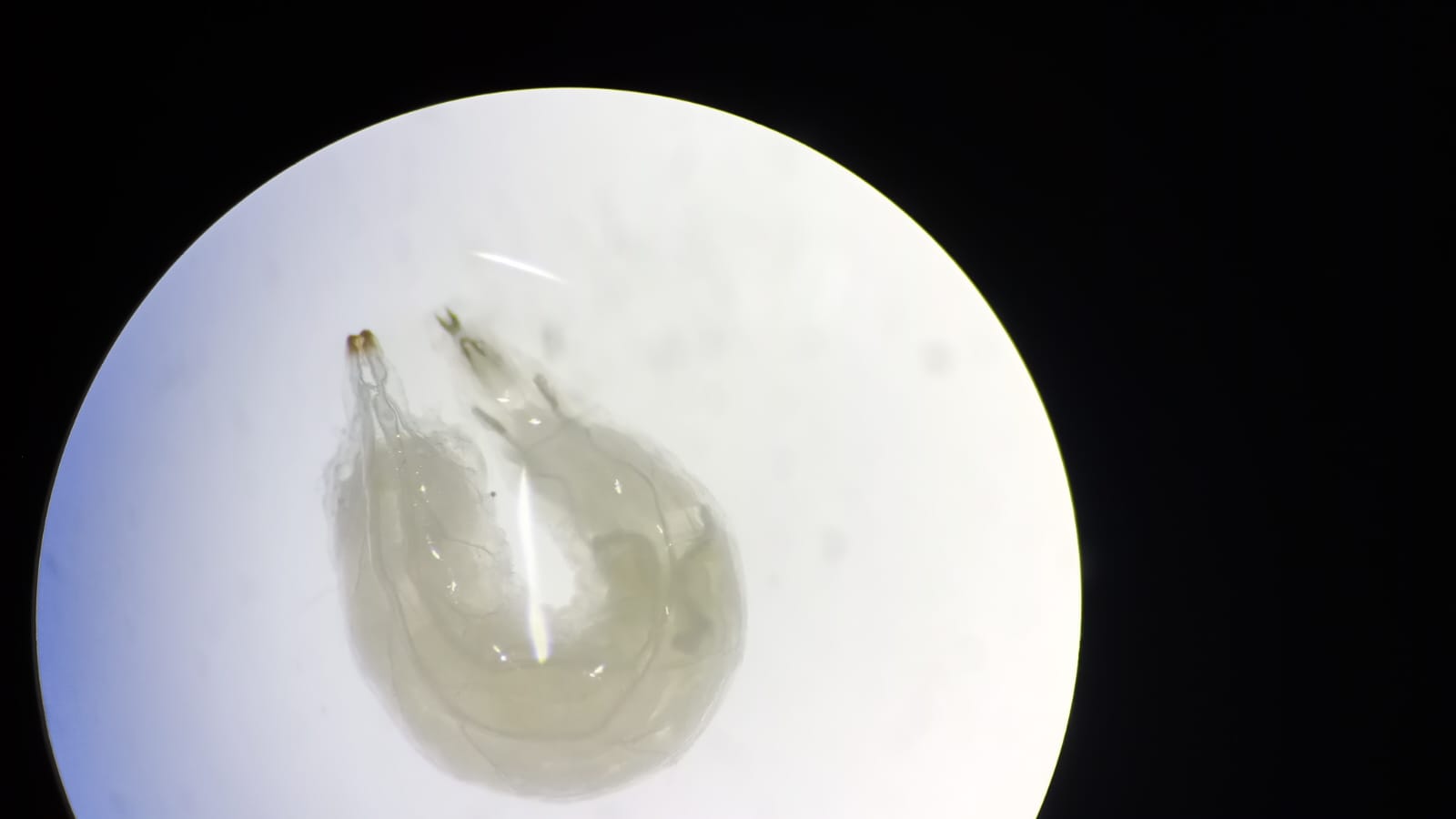


**A**


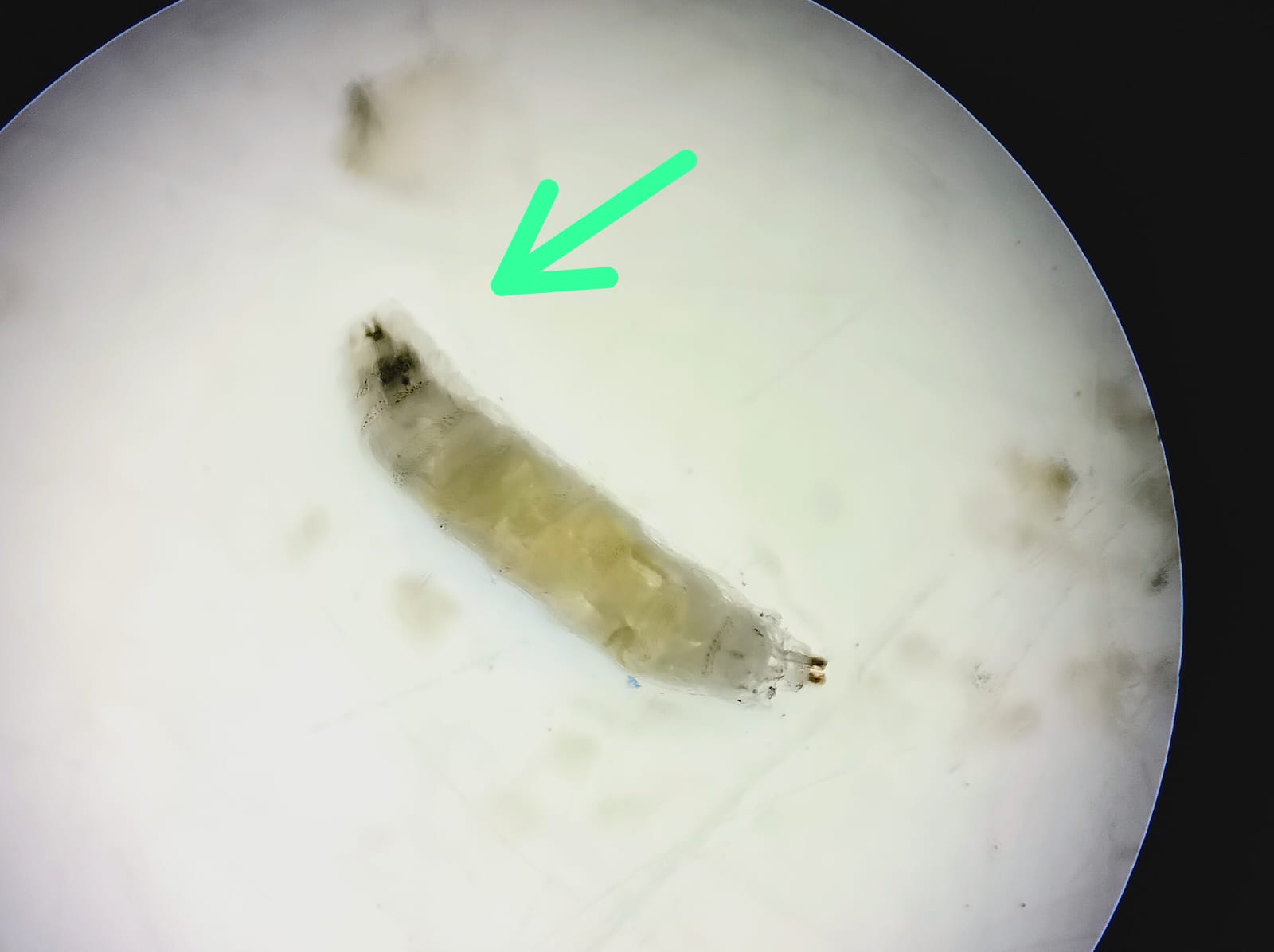


**H**


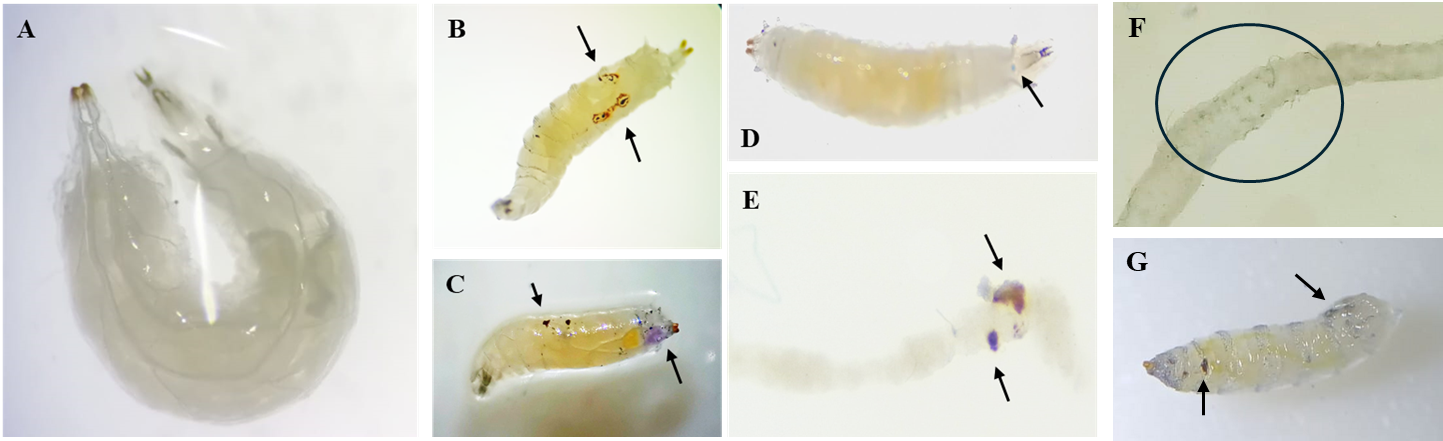

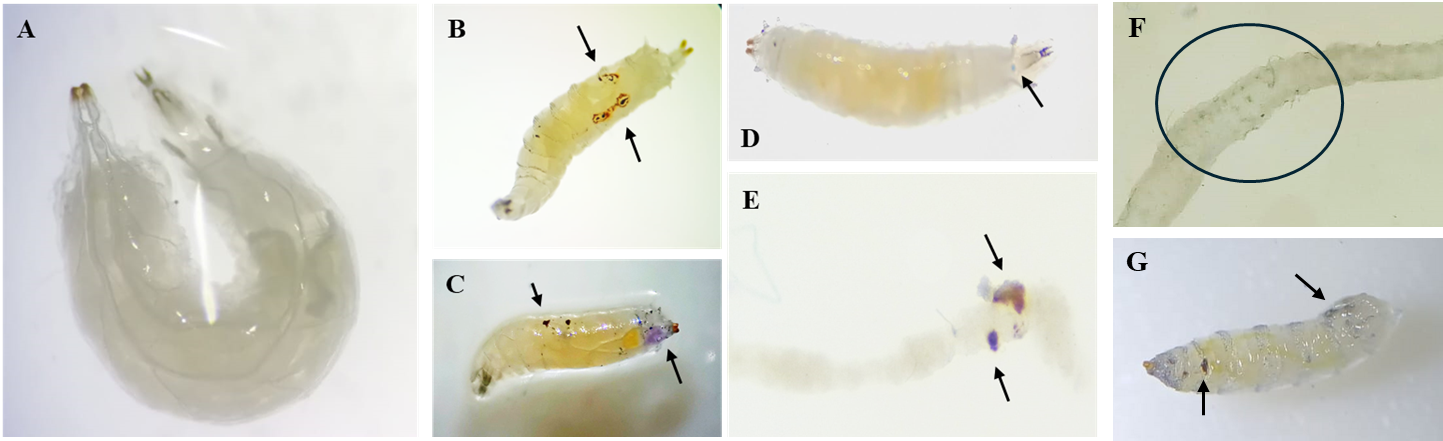


Fig 3S. Tissue damage in third-instar larvae of *Drosophila melanogaster*

(A) Negative control (4x). (B) Larvae exposed to 0.02% H₂O₂ showing tegument damage. (C) Larvae exposed to 50% ethanol showing damage to the tegument and posterior spiracles. (D–E) PP-MPs exposure showing minimal damage in the cephalic segment (D) and intestinal damage (E). (F–G) DMA exposure showing intestinal (F) and tegument damage (G). (H) Cephalic segment damage in larvae exposed to PP-MPs + DMA. Whole larvae were observed at 2× and intestines at 4× magnification.
